# Evaluation of the impact of glycemic status on the progression of coronary artery calcification in asymptomatic individuals

**DOI:** 10.1186/s12933-017-0653-0

**Published:** 2018-01-04

**Authors:** Ki-Bum Won, Donghee Han, Ji Hyun Lee, Sang-Eun Lee, Ji Min Sung, Su-Yeon Choi, Eun Ju Chun, Sung Hak Park, Hae-Won Han, Jidong Sung, Hae Ok Jung, Hyuk-Jae Chang

**Affiliations:** 1 Division of Cardiology, Ulsan University Hospital, University of Ulsan College of Medicine, Ulsan, South Korea; 2 Division of Cardiology, Yonsei Cardiovascular Center, Yonsei University Health System, Seoul, South Korea; 3 Division of Cardiology, Healthcare System Gangnam Center, Seoul National University Hospital, Seoul, South Korea; 4 Division of Radiology, Seoul National University Bundang Hospital, Seongnam, South Korea; 5 Division of Radiology, Gangnam Heartscan Clinic, Seoul, South Korea; 6 Department of Internal Medicine, Gangnam Heartscan Clinic, Seoul, South Korea; 7 Division of Cardiology, Heart Stroke & Vascular Institute, Samsung Medical Center, Seoul, South Korea; 8 Division of Cardiology, Department of Internal Medicine, College of Medicine, Seoul St. Mary’s Hospital, The Catholic University of Korea, Seoul, South Korea; 9 Division of Cardiology, Severance Cardiovascular Hospital, Yonsei University College of Medicine, Yonsei University Health System, 50-1 Yonsei-ro, Seodaemun-gu, Seoul, 03722 South Korea

**Keywords:** Pre-diabetes, Diabetes, Coronary artery calcification

## Abstract

**Background:**

Data on the influence of glycemic status on the progression of coronary calcification, an important marker for future adverse cardiovascular events, are limited.

**Methods:**

Data from the Korea Initiatives on Coronary Artery Calcification (KOICA) registry on 12,441 asymptomatic Korean adults (52 ± 9 years, 84.2% males) without previous history of coronary artery disease and stroke, who underwent serial coronary artery calcification (CAC) screening examinations, were included in this study. The median inter-scan period was 3.0 (2.0–4.8) years. All participants were categorized into three groups based on their glycemic status: normal (n = 6578), pre-diabetes (n = 4146), and diabetes (n = 1717). CAC progression was defined as a difference ≥ 2.5 between the square roots (√) of the baseline and follow-up CAC scores.

**Results:**

The incidence of CAC progression was significantly different between the three groups (normal, 26.3%; pre-diabetes, 30.9%; and diabetes, 46.9%; p < 0.001). In the univariate logistic analysis, the risk of CAC progression was higher in the pre-diabetes (odds ratio [OR] 1.253; 95% confidential interval [CI] 1.150–1.366) and diabetes (OR 2.471; 95% CI 2.215–2.758) groups than in the normal group (p < 0.001, both). In the multivariate logistic analysis, the risk of CAC progression was not significantly different between the normal and pre-diabetes groups but was significantly higher in the diabetes group than in the normal group.

**Conclusions:**

In asymptomatic subjects, diabetes had an incremental impact on CAC progression; however, pre-diabetes did not increase the risk of CAC progression after adjusting for confounding factors.

## Background

Diabetes is one of the major causes of cardiovascular (CV) morbidity and mortality worldwide. Several meta-analyses have reported that diabetes increases the risk of developing coronary artery disease (CAD) two to threefold [1–4]. It is well-known that diabetes is associated with an increased risk of CAD after adjusting for other traditional CV risk factors. However, uncertainty remains as to whether a pre-diabetic condition is independently associated with the progression of coronary atherosclerosis [5, 6].

Coronary artery calcification (CAC) is associated with atherosclerotic burden and adverse CV clinical outcomes [7–10]. Furthermore, CAC progression is a powerful predictor of mortality over the baseline CAC score and traditional CV risk factors [11]. Therefore, this study aimed to explore the association between glycemic status and CAC progression in an asymptomatic Korean population using serial cardiac computed tomography (CT) scans.

## Methods

### Study population and design

Data from the Korea Initiatives on Coronary Artery Calcification (KOICA) multicenter registry were analyzed. This is a retrospective, single ethnicity, multicenter observational registry in a self-referral setting for subjects who underwent health checkups at six health care centers in South Korea. A total of 93,707 subjects were enrolled in the KOICA registry from December 2012 to August 2016. Self-reported medical questionnaires were used to obtain information about medical history. All data were obtained during visits to each healthcare center. Among the 93,707 subjects from this registry, 12,441, who underwent at least two CAC scan examinations with available glycemic status data, were included in this study. All participants were categorized into three groups based on their glycemic status: normal, pre-diabetes, and diabetes. Pre-diabetes was defined as a fasting plasma glucose (FPG) level of 100–125 mg/dL or hemoglobin A1c (HbA1c) levels of 5.7–6.4% [12]. Diabetes was defined as either an FPG level of ≥ 126 mg/dL, HbA1c level of ≥ 6.5%, a referral diagnosis of diabetes, or currently receiving anti-diabetic treatment [12, 13].

Coronary artery calcification score was determined based on the scoring system previously described by Agatston et al. [14]. CAC progression was defined as a difference ≥ 2.5 between the square roots (√) of the baseline and follow-up CAC scores (Δ √transformed CAC) [15]. The appropriate institutional review board committees of each healthcare center approved the study protocol. Information on the medical history of hypertension, diabetes, and smoking status for each subject was systematically collected. Height, weight, and blood pressure were measured during the healthcare center visits. All blood samples were obtained after a minimum of an 8 h fast and analyzed for triglyceride, high-density lipoprotein (HDL) cholesterol, low-density lipoprotein (LDL) cholesterol, and glucose levels. In all centers, a CT scan to assess CAC was performed using a > 16 slice multi-detector CT scanner (Siemens 16-slice Sensation, Philips Brilliance 256 iCT, Philips Brilliance 40 channel MDCT, and GE 64-slice Lightspeed). All centers performed standard prospective or retrospective methods.

### Statistical analysis

Continuous variables are expressed as mean ± standard deviation. Categorical variables are presented as absolute values and proportions. To compare the characteristics of participants among the three glycemic groups, the one-way analysis of variance with Bonferroni’s post hoc test or the Kruskal–Wallis test was used for continuous variables, as appropriate, and the Chi square test for categorical variables. Univariate logistic regression analysis was performed to identify the clinical factors significant for CAC progression. Subsequently, multivariate logistic regression analysis was used to identify the independent impact of the glycemic status on CAC progression after adjusting the variables with p < 0.05 in the univariate analysis. All statistical analyses were performed using the Statistical Package for the Social Sciences version 19 (SPSS, Chicago, Illinois), and a p value of < 0.05 was considered significant for all analyses.

## Results

### Baseline characteristics

The mean age of participants was 52 ± 9 years, and a total of 10,472 (84%) participants were males. Among them, 6578 (52.9%), 4146 (33.3%), and 1717 (13.8%) were categorized in normal, pre-diabetes, and diabetes groups, respectively. Table 1 describes the baseline characteristics of participants based on their glycemic status. The clinical characteristics such as age, anthropometric indices, including body mass index and waist circumference, and the incidence of hypertension, dyslipidemia, and smoking were significantly different among all groups.

**Table 1:** Baseline characteristics

	Normal (n = 6578)	Pre-diabetes (n = 4146)	Diabetes (n = 1717)	p
Age, years	50 ± 8	53 ± 8*	55 ± 9*^†^	< 0.001
Male, n (%)	5423 (82.4)	3523 (85.0)	1526 (88.9)	< 0.001
Body mass index, kg/m^2^	24.1 ± 2.6	25.0 ± 2.8*	25.2 ± 2.9*^†^	< 0.001
Waist circumference, cm	85 ± 8	88 ± 8*	89 ± 8*^†^	< 0.001
Systolic blood pressure, mmHg	118 ± 15	122 ± 15*	122 ± 16*	< 0.001
Diastolic blood pressure, mmHg	74 ± 11	77 ± 10*	76 ± 10*	< 0.001
Hypertension, n (%)	1568 (24.4)	1544 (39.1)	924 (55.1)	< 0.001
Antihypertensive drugs, n (%)	984 (17.0)	1077 (30.2)	675 (43.2)	< 0.001
Dyslipidemia, n (%)	1481 (22.6)	1286 (31.0)	701 (40.8)	< 0.001
Lipid lowering drugs, n (%)	89 (4.2)	143 (10.8)	76 (18.8)	< 0.001
Smoking, n (%)	3919 (64.4)	2552 (68.8)	1166 (74.0)	< 0.001
Total cholesterol, mg/dL	196 ± 33	203 ± 34*	190 ± 36*^†^	< 0.001
Triglyceride, mg/dL	130 ± 79	153 ± 96*	156 ± 102*	< 0.001
HDL cholesterol, mg/dL	54 ± 16	53 ± 16*	51 ± 16*^†^	< 0.001
LDL cholesterol, mg/dL	122 ± 31	125 ± 33*	115 ± 33*^†^	< 0.001
Creatinine, mg/dL	0.9 ± 0.2	1.0 ± 0.2	1.0 ± 0.2	0.096
hs-CRP, mg/dL	0.3 ± 1.5	0.4 ± 2.1	0.3 ± 1.7	0.086
Fasting glucose, mg/dL	88 ± 7	100 ± 10*	129 ± 35*^†^	< 0.001
HbA1C, %	5.3 ± 0.3	5.7 ± 0.3*	6.8 ± 1.1*^†^	< 0.001

Values are given as mean ± standard deviation or number (%)

*HDL* high-density lipoprotein, *hs-CRP* high-sensitivity C-reactive protein, *LDL* low-density lipoprotein

* p < 0.05 vs. normal, ^†^ p < 0.05 vs. pre-diabetes

### CAC changes based on the glycemic status

**Fig. 1:**
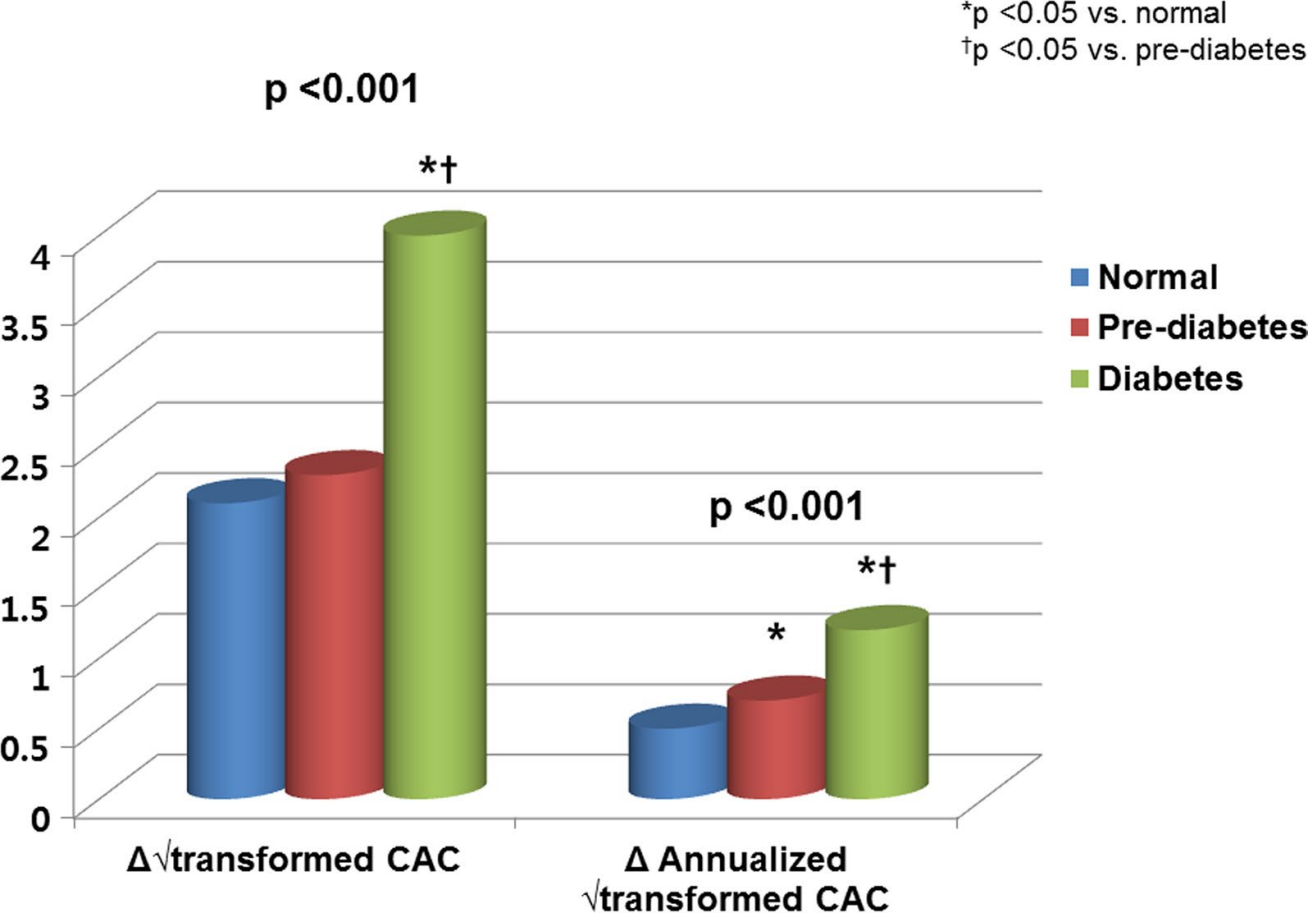
Change of CAC according to glycemic control status

Table 2 presents the baseline and follow-up CAC scores based on the glycemic status. The average inter-scan period was 3.3 ± 1.8 years. The baseline and follow-up CAC scores based on glycemic status were significantly different (p < 0.001 for all). The incidence of CAC progression was significantly different among the three groups (normal, 26.3%; pre-diabetes, 30.9%; diabetes, 46.9%; p < 0.001). Both the Δ √transformed CAC (normal, 2.1 ± 4.4; pre-diabetes, 2.3 ± 4.3; diabetes, 4.0 ± 6.5; p < 0.001) and the annualized Δ √transformed CAC (normal, 0.5 ± 1.6; pre-diabetes, 0.7 ± 1.6; diabetes, 1.2 ± 2.4; p < 0.001) scores were different among the three groups. The diabetes group had higher Δ √transformed CAC and annualized Δ √transformed CAC scores than the pre-diabetes and normal groups (p < 0.05, respectively). The Δ √transformed CAC between normal and pre-diabetes groups were not significantly different. However, the pre-diabetes group had higher annualized Δ √transformed CAC values than the normal group (Fig. 1).

**Table 2:** Progression of CAC based on the glycemic status

	Normal (n = 6578)	Pre-diabetes (n = 4146)	Diabetes (n = 1717)	p
Baseline				
CAC score	28 ± 110	49 ± 158*	110 ± 319*^†^	< 0.001
Categorical CAC score				< 0.001
0	4308 (65.5)	2109 (50.9)	554 (32.3)	
1–10	840 (12.8)	608 (14.7)	286 (16.7)	
11–100	1002 (15.2)	924 (22.3)	490 (28.5)	
101–400	324 (4.9)	407 (9.8)	266 (15.5)	
> 400	104 (1.6)	98 (2.4)	121 (7.0)	
Follow-up				
CAC score	71 ± 209	97 ± 235*	220 ± 422*^†^	< 0.001
Categorical CAC score				< 0.001
0	3657 (55.6)	1732 (41.8)	411 (23.9)	
1–10	550 (8.4)	390 (9.4)	122 (7.1)	
11–100	1321 (20.1)	1048 (25.3)	491 (28.6)	
101–400	751 (11.4)	712 (17.2)	410 (23.9)	
> 400	299 (4.5)	264 (6.4)	283 (16.5)	
Inter-scan period (years)	3.1 (2.0–5.0)	3.0 (1.9–4.5)	3.0 (2.0–4.3)	< 0.001
CAC progression, n (%)	1731 (26.3)	1282 (30.9)	805 (46.9)	< 0.001

*CAC* coronary artery calcium

* p < 0.05 vs. normal, ^†^ p < 0.05 vs. pre-diabetes

### Association between clinical factors and CAC progression

Univariate logistic regression analysis showed that age (odds ratio [OR] 1.070; 95% confidential interval [CI] 1.065–1.076), male gender (OR 2.650; 95% CI 2.334–3.010), body mass index (OR 1.093; 95% CI 1.078–1.108), hypertension (OR 2.091; 95% CI 1.930–2.266), dyslipidemia (OR 1.738; 95% CI 1.600–1.887), smoking (OR 1.718; 95% CI 1.571–1.879), and baseline CAC score > 100 (OR 2.818; 95% CI 2.510–3.164) were significantly associated with CAC progression (p < 0.001, respectively). Compared with the normal group, the subjects in the pre-diabetes (OR 1.253; 95% CI 1.150–1.366) and diabetes (OR 2.471; 95% CI 2.215–2.758) groups had an increased risk of CAC progression (p < 0.001, respectively). Multivariate logistic regression analysis showed that age (OR 1.069; 95% CI 1.063–1.075), male gender (OR 2.422; 95% CI 2.056–2.853), body mass index (OR 1.061; 95% CI 1.043–1.079), hypertension (OR 1.364; 95% CI 1.243–1.498), dyslipidemia (OR 1.464; 95% CI 1.335–1.606), smoking (OR 1.386; 95% CI 1.247–1.540), and baseline CAC score > 100 (OR 1.321; 95% CI 1.153–1.513) were significantly associated with CAC progression (p < 0.001, respectively). Compared with the normal group, the risk of CAC progression was not significantly different in pre-diabetes group (OR 0.943; 95% CI 0.856–1.040), but was significantly higher in the diabetes group (OR 1.368; 95% CI 1.206–1.553) (Table 3).

**Table 3:** Logistic regression models to identify independent predictors of CAC progression

Variables	Univariate		Multivariate	
	OR (95% CI)	p	OR (95% CI)	p
Age, years	1.070 (1.065–1.076)	< 0.001	1.069 (1.063–1.075)	< 0.001
Male gender	2.650 (2.334–3.010)	< 0.001	2.422 (2.056–2.853)	< 0.001
Body mass index, kg/m^2^	1.093 (1.078–1.108)	< 0.001	1.061 (1.043–1.079)	< 0.001
Hypertension	2.091 (1.930–2.266)	< 0.001	1.364 (1.243–1.498)	< 0.001
Dyslipidemia	1.738 (1.600–1.887)	< 0.001	1.464 (1.335–1.606)	< 0.001
Smoking	1.718 (1.571–1.879)	< 0.001	1.386 (1.247–1.540)	< 0.001
Baseline CAC score > 100	2.818 (2.510–3.164)	< 0.001	1.321 (1.153–1.513)	< 0.001
Glycemic status				
Normal	1	–	1	–
Pre-diabetes	1.253 (1.150–1.366)	< 0.001	0.943 (0.856–1.040)	0.241
Diabetes	2.471 (2.215–2.758)	< 0.001	1.368 (1.206–1.553)	< 0.001

*CAC* coronary artery calcium, *CI* confidence interval, *OR* odds ratio

### Independent predictors for CAC progression according to glycemic status

The results of multivariate logistic regression analysis to identify the independent predictors for CAC progression according to glycemic status are presented in Table 4. In the normal group, age, male gender, body mass index, hypertension, dyslipidemia, smoking, and baseline CAC score > 100 were significantly associated with CAC progression. All factors, except baseline CAC score > 100, were also associated with CAC progression in the pre-diabetes group. However, only age and male gender were independent predictors of CAC progression in the diabetes group.

**Table 4:** Independent predictors for CAC progression according to glycemic status

Variables	Normal		Pre-diabetes		Diabetes	
	OR (95% CI)	p	OR (95% CI)	p	OR (95% CI)	p
Age, years	1.090 (1.080–1.099)	< 0.001	1.057 (1.047–1.068)	< 0.001	1.036 (1.022–1.050)	< 0.001
Male gender	2.447 (1.921–3.118)	< 0.001	2.450 (1.857–3.231)	< 0.001	1.895 (1.279–2.810)	0.001
Body mass index, kg/m^2^	1.073 (1.045–1.102)	< 0.001	1.051 (1.022–1.081)	< 0.001	1.035 (0.997–1.074)	0.069
Hypertension	1.478 (1.284–1.701)	< 0.001	1.552 (1.332–1.808)	< 0.001	0.910 (0.732–1.131)	0.395
Dyslipidemia	1.658 (1.443–1.905)	< 0.001	1.590 (1.365–1.853)	< 0.001	0.980 (0.794–1.209)	0.849
Smoking	1.463 (1.256–1.703)	< 0.001	1.385 (1.155–1.660)	< 0.001	1.170 (0.900–1.523)	0.241
Baseline CAC score > 100	1.685 (1.333–2.131)	< 0.001	1.212 (0.974–1.508)	0.085	1.286 (0.994–1.663)	0.056

*CAC* coronary artery calcium, *CI* confidence interval, *OR* odds ratio

## Discussion

The main finding of this longitudinal study is that diabetes has an independent impact on CAC progression; however, pre-diabetes is not associated with CAC progression after adjusting for confounding clinical factors in asymptomatic subjects.

Coronary artery calcification is an important marker to identify the presence of CAD in asymptomatic subjects. It is a well-known and useful tool to predict adverse clinical events [7–10]. Previous cross-sectional studies reported the significant risk factors for CAC [16–18].

Recently, the emphasis has been on identifying CAC progression in clinical practice because of its increasing value in predicting adverse outcomes over the baseline CAC score, time between scans, demographics, and CV risk factors [11]. Several previous studies have investigated the predictive value of clinical factors for CAC progression. In the Heinz Nixdorf Recall study, CAC inevitably progressed with limited influence of CV risk factors [19]. Diederichsen et al. [20] recently evaluated the predictive value of 15 biomarkers for CAC incidence and progression in asymptomatic middle-aged subjects at 5 years follow-up. This prospective study identified the association between total and LDL cholesterol and CAC incidence, and between phosphate and CAC progression; however, 12 other biomarkers did not significantly correlate with CAC. Nevertheless, the impact of glycemic status, especially in a pre-diabetic condition, on CAC progression in asymptomatic individuals has remained uncertain.

In the Multi-Ethnic Study of Atherosclerosis (MESA) study, diabetes had the strongest impact on CAC progression in Black, and the weakest in Hispanic people, with intermediate associations in White and Chinese people [21]. Furthermore, Wong et al. [22] reported that subjects with metabolic syndrome (MetS) and diabetes had a greater CAC incidence and absolute progression compared with those without these conditions. However, the proportion of Asian participants in the study was small, and the association between pre-diabetes and CAC progression was not evaluated in the MESA study. Other studies have evaluated the relationship between pre-diabetes and the risk of CAD; however, the results were inconsistent [5, 23].

Recently, a large observational study reported that pre-diabetes was not associated with an increased risk of subclinical coronary atherosclerosis in an asymptomatic Korean population [24]. However, this study did not evaluate the atherosclerotic progression because of its cross-sectional design. The result of the present study is consistent in that pre-diabetes did not have an independent impact on CAC progression. Contrary to these results, Lee et al. [25] reported that MetS influenced the progression of CAD as assessed by CAC scores and coronary CT angiography in a relatively healthy Korean population. However, this study included established diabetes in the definition of MetS. Considering that the World Health Organization recommends that the concept of MetS should not be applied in subjects with established diabetes [26], pre-diabetes is different from MetS in subjects without established diabetes. Thus, the emphasis should not only be on controlling concomitant metabolic risk factors but also on preventing the development of diabetes to inhibit CAC progression in asymptomatic pre-diabetics in clinical practice.

The present study identified that diabetes was associated with an increased risk of CAC progression after adjusting for confounding factors. It is well-known that vascular calcification is increased in diabetes, and the presence of CAC is a strong risk factor for CV events [27]. One of the most important mechanisms in diabetes patients was hyperglycemic damage, mainly driven by the accumulation of free radicals, which activates vascular inflammation and endothelial dysfunction. In addition, hyperglycemia itself also increases oxidative stress by increasing glucose oxidation [28]. Previous studies strongly suggested that hyperglycemia may directly influence the atherosclerotic process and prognosis in established diabetes patients [29–33]. In contrast, several studies reported that MetS does not significantly influence subclinical atherosclerosis and mortality in relatively healthy diabetes patients [34, 35]. In the present study, we observed that traditional CV risk factors, such as hypertension, dyslipidemia, and smoking, were independent determinants for CAC progression in both normal and pre-diabetes, but were not associated with CAC progression in diabetes patients. Thus, strict glycemic control may be more important to prevent future CV events in asymptomatic subjects with diabetes than traditional risk factors. Further investigation to identify the impact of strict glycemic control on CAC progression and prognosis is necessary in asymptomatic patients with established diabetes.

Although multiple imaging techniques are available to detect the presence of pre-clinical disease, the efficacy of these modalities in the risk stratification of asymptomatic diabetes patients remains uncertain. Recently, Rassi et al. [36] reported that CAC was the most accurate screening modality for detection of CAD, but that screening for carotid plaque using normal coronary CT angiography may better characterize stroke risk in asymptomatic diabetics. However, this study was performed in only 98 participants. Further prospective studies with larger sample sizes are required to address this issue.

The present study has several limitations. First, the KOICA registry was based on a healthy population who underwent health checkups in healthcare centers, which may result in a potential selection bias. Second, this was a retrospective study, which may be influenced by unidentified confounders. Third, participants did not undergo an oral glucose tolerance test; however, FPG is proven to be a useful parameter in clinical practice [37]. Fourth, data on the physical activity of participants were unavailable. However, this study was performed on asymptomatic subjects in a self-referral setting. Fifth, we could not eliminate the possible effects of medications for hypertension, dyslipidemia, and diabetes on the progression of CAC because of the observational design. Further large prospective studies are necessary to address these issues. Finally, the present study included the Korean population only. However, this longitudinal study uniquely identified the CAC progression based on the glycemic status in asymptomatic individuals, specifically in the Asian population.

## Conclusion

In conclusion, diabetes had an incremental effect on CAC progression; however, pre-diabetes was not associated with an increased risk of CAC progression after adjusting for confounding factors in asymptomatic subjects.

## Abbreviations

CAC: coronary artery calcification
CAD: coronary artery disease
CI: confidence interval
CT: computed tomography
FPG: fasting plasma glucose
HbA1c: hemoglobin A1c
HDL: high-density lipoprotein
KOICA: Korea Initiatives on Coronary Artery Calcification
LDL: low-density lipoprotein
MESA: Multi-Ethnic Study of Atherosclerosis
OR: odds ratio
